# The Impact of Nucleos(t)ide Analogs Off-Therapy Among Chronic Hepatitis B Patients: A Systematic Review and Meta-Analysis

**DOI:** 10.3389/fpubh.2021.709220

**Published:** 2021-09-10

**Authors:** Mian Wang, Mingxia Qian, Rongrong Fu, Yiqin Zhang, Xinlan Shen, Dengyuan Yue, Ning Wang, Lei Yang

**Affiliations:** ^1^Infection Department, Ningbo Yinzhou No. 2 Hospital, Ningbo, China; ^2^School of Public Health, Zhejiang Chinese Medical University, Hangzhou, China; ^3^The First Clinical Medical College, Zhejiang Chinese Medical University, Hangzhou, China; ^4^Emergency Medical Center, Ningbo Yinzhou No. 2 Hospital, Ningbo, China; ^5^The Second Clinical Medical College, Zhejiang Chinese Medical University, Hangzhou, China

**Keywords:** chronic hepatitis B, off-treatment, maintained, nucleos(t)ide analogs, meta-analysis

## Abstract

**Background and Aim:** Although most chronic hepatitis B (CHB) patients achieve effective virological suppression after receiving long-term nucleos(t)ide analogs (Nucs) therapy, the safety of off-therapy is controversial under the monitor.

**Methods:** We identified studies through searching PubMed, Embase, Cochrane Library, and Web of Science from January 1990 to February 2021. The eligible studies compare the long outcomes between discontinued and continued Nucs treatments groups among CHB patients. This study was conducted to investigate long-term outcomes, including biochemical, serological, and virological outcomes, as well as hepatocellular carcinoma (HCC) development rate between discontinued and maintained Nucs therapy groups among CHB patients.

**Results:** Five eligible studies covering 1,425 patients were selected for meta-analysis. Our result exhibits that patients with Nucs off-treatment have a higher risk of alanine aminotransferase (ALT) flares-up than those who continued Nucs therapy under the monitor (OR = 9.39, 95%CI = 3.87–22.78). Nucs off-therapy patients have a higher virological bound incidence (OR = 617.96, 95%CI = 112.48–3,395.14) and a higher HBV DNA level (OR = 9.39, 95%CI = 3.87–22.78) than those who continued Nucs therapy. There was no statistically significant difference in the risk of hyperbilirubinaemia, hepatic decompensation, and HCC development between both two groups. Patients in Nucs off-therapy group demonstrate a higher HBsAg loss rate than those in the continued group (OR = 7.10, 95%CI = 6.68–13.69).

**Conclusions:** Nucs off-therapy patients may exhibit a higher chance of achieving HBsAg loss than those who continue Nucs therapy. It requires close monitoring after Nucs off-therapy and timely restarting of Nucs therapy when ALT concentrations increase.

## Introduction

Over 257 million people, or 3.2% of the global population, are estimated to have chronic hepatitis B infection (CHB) (1, 2). Although there is currently no cure for hepatitis B virus (HBV) infection, it can be effectively controlled with existing antiviral treatment strategies utilizing either interferon (IFN) or nucleos(t)ide analogs (Nucs) (1, 2). Nucs therapy for CHB patients works by suppressing HBV replication over time, thus preventing disease progression to decompensated cirrhosis and hepatocellular carcinoma (HCC) (3–7). HBeAg seroconversion is the ideal endpoint for treating hepatitis B “e” antigen (HBeAg)-positive patients; this is often accompanied by hepatitis B surface antigen (HBsAg) loss, which is thought to be the nearest to therapeutic cure of chronic HBV infection. In contrast, the only endpoint defined for HBeAg-negative patients is HBsAg loss (8). However, these endpoints are seldom reached with current methods, and long-term Nucs therapy poses many concerns, including adherence, compliance issues, and, most significantly, costs (9–11). As a result, studies are being conducted to determine whether Nucs off-therapy can substitute lifelong therapy in CHB patients who are closely monitored (12, 13).

Although previous studies indicated that Nucs off-therapy would result in a series of adverse outcomes such as virological relapse (14) and alanine aminotransferase (ALT) flares-up (15), some recent studies (13) demonstrated that Nucs off-therapy patients might experience HBsAg loss than those who continued Nucs therapy under the monitor. Therefore, Nucs off-therapy is an intriguing approach to explore and investigate further. Simultaneously, there is a need to further compare the risk of clinical complications, particularly liver cirrhosis and HCC, in CHB patients treated with or without Nucs therapy. Notably, Nucs off-therapy in liver cirrhosis patients might even increase the risk of long-term adverse outcomes such as HCC and mortality (16).

To resolve these controversies, we conducted a comprehensive meta-analysis pooling the available data to compare discontinued and maintained Nucs therapy in CHB patients to provide a practical and safe approach for the optimal management of CHB patients.

## Methods

### Search Strategy

We identified studies through searching PubMed, Embase, Cochrane Library, and Web of Science from January 1990 to February 2021 under the search text terms “chronic hepatitis B” or “HBV” and “nucleos(t)ide analogs” or “NAs” or “Nucs” or “lamivudine” or “adefovir” or “entecavir” or “telbivudine” or “tenofovir” and “interruption” or “stop” or “discontinuation” or “off-therapy” or “off-treatment” or “end” or “withdrawal” or “cessation.” Moreover, relevant references in literature were manually searched to avoid omitting studies.

### Selection Criteria

Two independent authors retrieved the studies according to inclusion and exclusion criteria. In our study, published full papers were eligible for inclusion if they met the following criteria: (1) Randomized controlled trials (RCTs) or observational studies (cohort or case-control). (2) Patients with chronic hepatitis B. (3) Articles compared between discontinued and continued Nucs treatments in CHB patients. (4) Available outcomes, including biochemical, serological, and virological outcomes, as well as HCC development rate. Exclusion criteria were formulated as follows: (1) Subjects with other causes of hepatitis, such as hepatitis C or hepatitis D. (2) Articles without relevant outcomes. (3) Duplicate literature. However, for duplicate articles, the literature with the newest or comprehensive data was included.

### Data Extraction and Study Quality Assessment

Two authors worked independently to extract data from the included papers and determine study quality assessment using a standardized form. If there is any uncertainty of a study between the two authors, it would be resolved by agreement between them or by the third reviewer. The database was used to record the available information, including author, year of publication, country, sample size, time to recruit, follow-up time, and study design, Nuc-experienced, characteristics of patients, and baseline level of HBV DNA. This meta-analysis was conducted per the guidelines of the Preferred Reporting Items for Systematic Review and Meta-Analysis Protocols (PRISMA-P) 2015 statement. The quality of RCTs and observational studies was determined using Cochrane Collaborations's tool (17, 18) and Newcastle-Ottawa Quality Assessment Scale (NOS) checklist, respectively, by two independent authors (19, 20) This quality assessment tool focuses on eight items categorized in three groups (selection, comparability, and outcome) with a maximum number of nine stars. The articles with six or more stars were deemed to be of higher quality.

### Statistical Analysis

Odds ratio (OR), including 95% confidence interval (CI), was used to assess the comparison between discontinued and continued Nucs therapies. Revman 5.3 Software (RevMan, The Cochrane Collaboration) was used to perform the meta-analysis and evaluate heterogeneity between studies by Cochrane Q test and *p*-values. If *p* ≥ 0.1, the fixed-effects model is applied, the random-effects model is applied if *p* < 0.1. The Stata 12.0 Software (Stata, College Station) was utilized to evaluate sensitivity and publication bias of studies. Publication bias was evaluated using Begg's and Egger's tests, and *p* < 0.05 was considered statistically significant. Publication bias and sensitivity analysis would not be performed on analysis subgroup with <10 studies due to low sensitivity of qualitative and quantitative tests.

## Results

**Figure 1:**
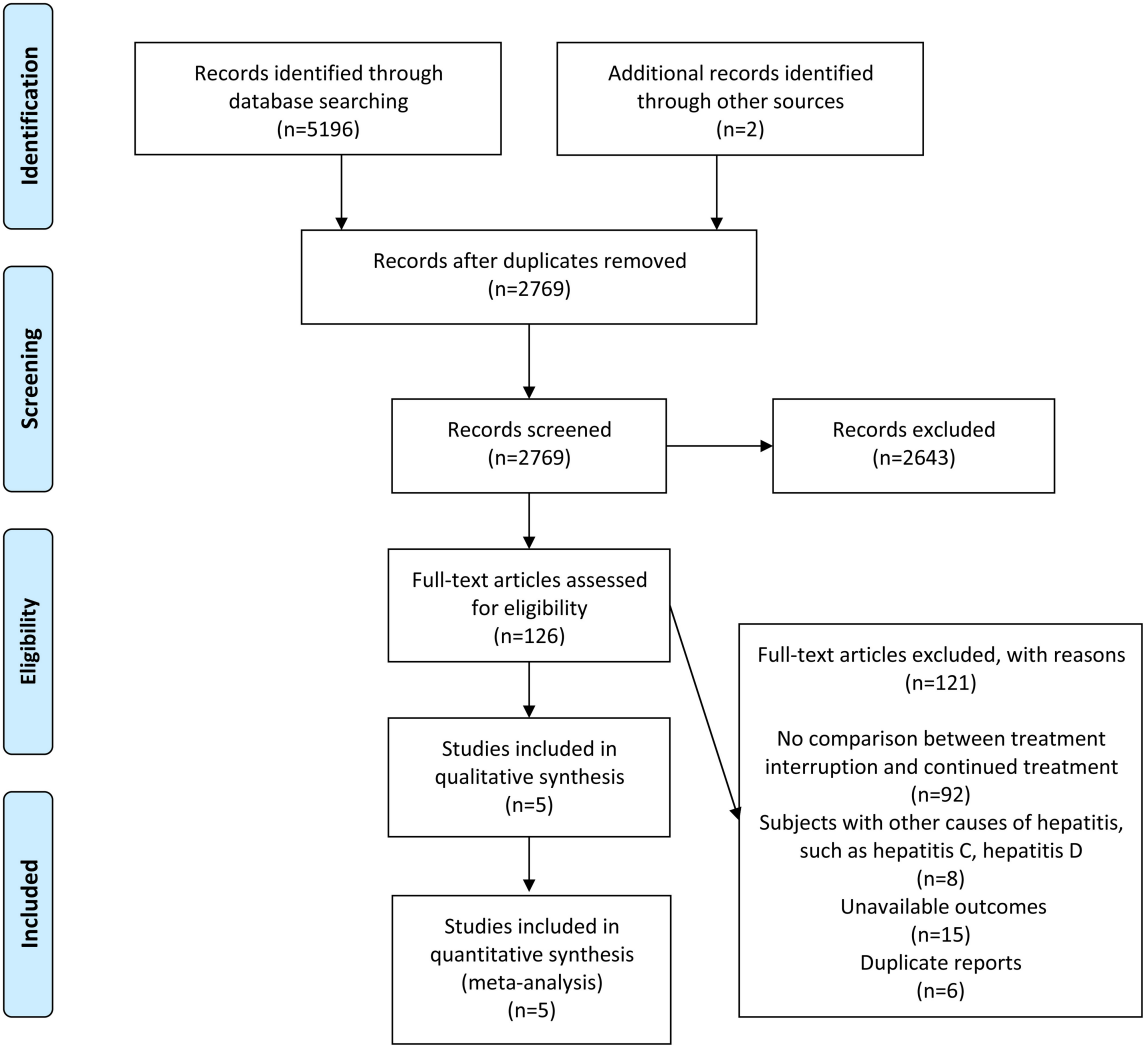
Flow diagram describing inclusion and exclusion criteria.

A total of 5,196 studies were derived from the search strategy, and two studies were identified through other sources. Of these, 2,429 studies were eliminated because of repetition, and 2,463 studies were excluded based on evaluation of their titles or abstracts, and the remaining 126 articles were scrutinized by full-text articles. Eventually, five eligible articles (13–15, 21, 22) were included in this meta-analysis, according to inclusion and exclusion criteria. The detailed search and study selection process is shown in Figure 1, and the quality of five studies was assessed as demonstrated in Supplementary Table 1.

### Study Characteristics

The characteristics of five studies with comparison between discontinued and continued Nucs treatments are displayed in Table 1. The five studies include four observational studies and one RCT, covering 1,452 CHB patients, comprising HBeAg-positive and HBeAg-negative patients. Among them, 554 patients conducted a Nucs off-treatment while the remaining 898 patients continued Nucs treatment. These CHB patients experienced Nucs therapy such as entecavir, lamivudine, and other Nucs. The primary outcomes are ALT flares-up, hyperbilirubinaemia level, hepatic decompensation, HBV DNA level, HBsAg loss, and HCC development.

**Table 1:** Characteristics of all the studies included in the meta-analysis.

**References**	**Country**	**Study**	**Time to recruit**	**Nuc-experienced (mean duration of treatment)**		**Number of patients**		**Characteristics of patients**		**HBV DNA at baseline**		**Length of follow-up**
				**Discontinued**	**Continued**	**Discontinued**	**Continuation**	**Discontinued**	**Continued**	**Discontinued**	**Continued**	
Fung et al. (22)	China	POBS	1994	Lamivudine; <10 years		22	79	HBeAg(+) CHB		8.6 (6.41–10.18) (log IU/ml)	7.87 (3.15–10.25) (log IU/ml)	5 years
Chen et al. (21)	China	POBS	NA	Entecavir; 30.3 ± 10.8 months	Entecavir; 56.9 ± 20.6 months	164	381	HBeAg(-) CHB with compensated cirrhosis		NA		60 months
Hung et al. (13)	China	POBS	2002	Nuc; <9 years		73	158	HBeAg(-) with cirrhosis	NA	5.20 ± 1.63 (log copies/mL)	6.07 ± 1.23 (log copies/mL)	>4 years
Liem et al. (15)	Canada	RCT	2016	Nuc; 7.6 ± 3.1 years	Nuc; 6.8 ± 2.4 years	45	22	HBeAg(-) n=27	HBeAg(-) n=13	6.1 ± 1.8 (log IU/ml)	6.0 ± 1.3 (log IU/mL)	72 weeks
Chen et al. (14)	China	POBS	NA	Nuc; 168.4 ± 41.4 weeks	0	250	231	HBeAg(-) without cirrhosis		<20 IU/ml		8 years

*NA, not available; RCT, randomized controlled trial; POBS, prospective observational study; Nuc, nucleos(t)ide analog; CHB, chronic hepatitis B; HBV, hepatitis B virus; HBeAg(+), hepatitis B e antigen positive; HBeAg(-), hepatitis B e antigen negative*.

### Biochemical Outcomes

In three studies involving 749 patients, 44 patients in the discontinued group underwent ALT flares-up whereas 13 patients in the continued group experienced ALT flares-up. By Fung et al. study, seven (32%) patients underwent biochemical ‘flares’ as defined by the elevation of ALT to twice the upper limit of normal (ULN), none experienced twice the upper limit of normal after continuation of NA therapy (22). By Liem et al. study, among patients who stopped therapy 14 (31%) developed ALT >10 ULN and another 7 (16%) patients had ALT >5 ULN (15). By Chen et al. study, in the discontinued group, 12 patients (3%) experienced severe ALT flare-up > 20 ULN after discontinuation of NA therapy, however, none experienced severe ALT flare-up > 20 ULN after continuation of NA therapy (14).

**Figure 2:**
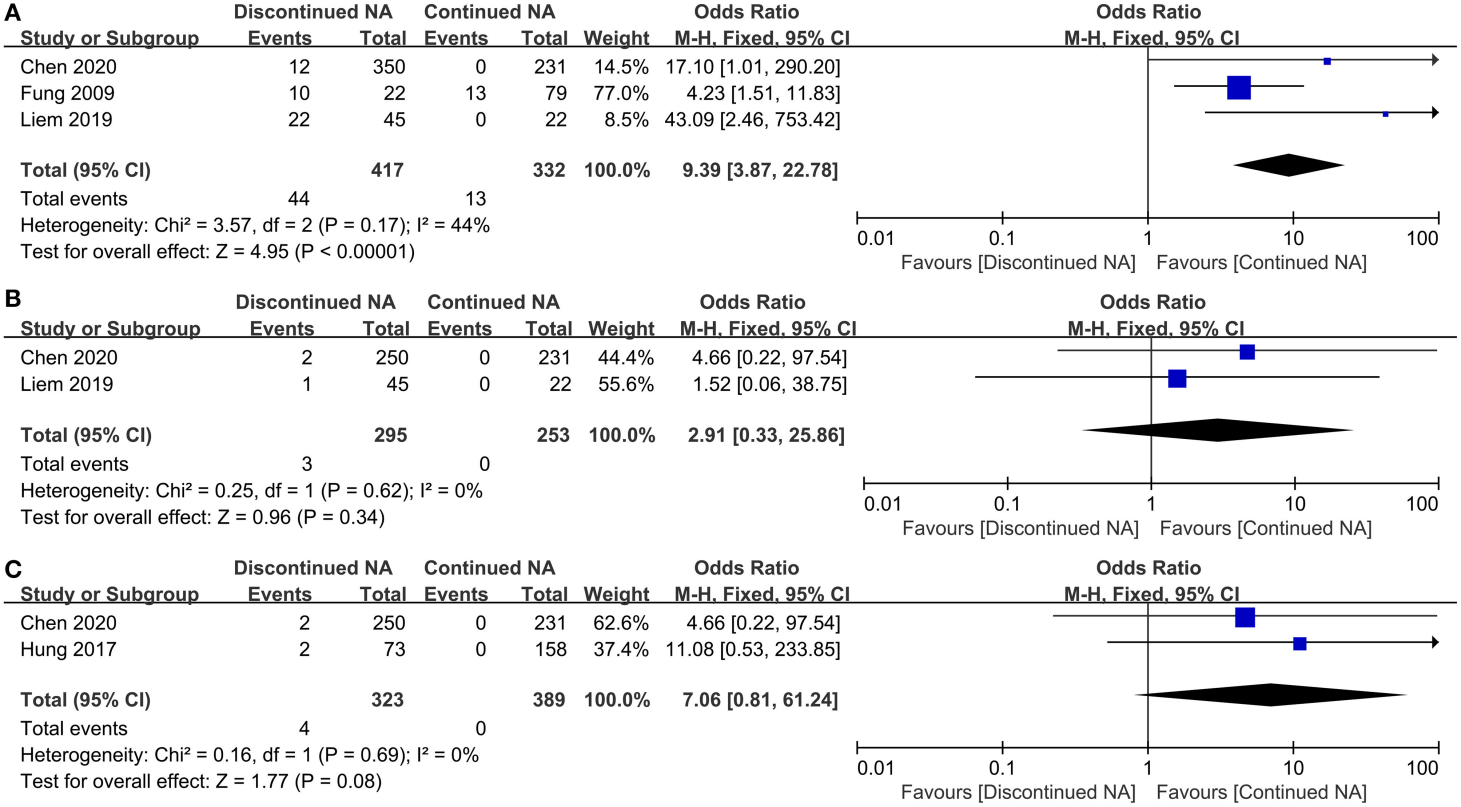
Forest plot on biochemical outcomes comparing between continued Nucs therapy and discontinued Nucs therapy. **(A)** ALT flares-up; **(B)** hyperbilirubinaemia level; **(C)** hepatic decompensation.

According to estimated pooled OR, our result exhibits that patients undergoing Nucs off-treatment have a higher risk of ALT flares-up compared with those who continued Nucs therapy (OR = 9.39, 95% CI = 3.87–22.78, *p* < 0.001, fixed-effects model) (Figure 2A). Additionally, two coincident studies (including 548 patients) investigated the increased risk of hyperbilirubinaemia level between off-therapy and continued groups. There was no statistically significant difference in the increased risk of hyperbilirubinaemia between discontinued and maintained Nucs groups (*p* = 0.34) (Figure 2B).

Only two studies were used to compare hepatic decompensation incidence among CHB patients between off-therapy and continued Nucs groups. In Nucs off-therapy group, four patients developed into hepatic decompensation, while no patients developed hepatic decompensation in the continued group. No statistically significant difference existed between the two groups in the incidence of hepatic decompensation (*p* = 0.08) (Figure 2C). Two patients (14) were not cirrhosis at baseline; NA treatment before treatment discontinuation is lasted 168.4 ± 41.4 weeks; follow-up is lasted 8 years. The others (13) were cirrhosis at baseline; NA treatment before treatment discontinuation is lasted <9 years; duration of follow-up is lasted >4 years.

### Virological Outcomes and Serological Outcomes

**Figure 3:**
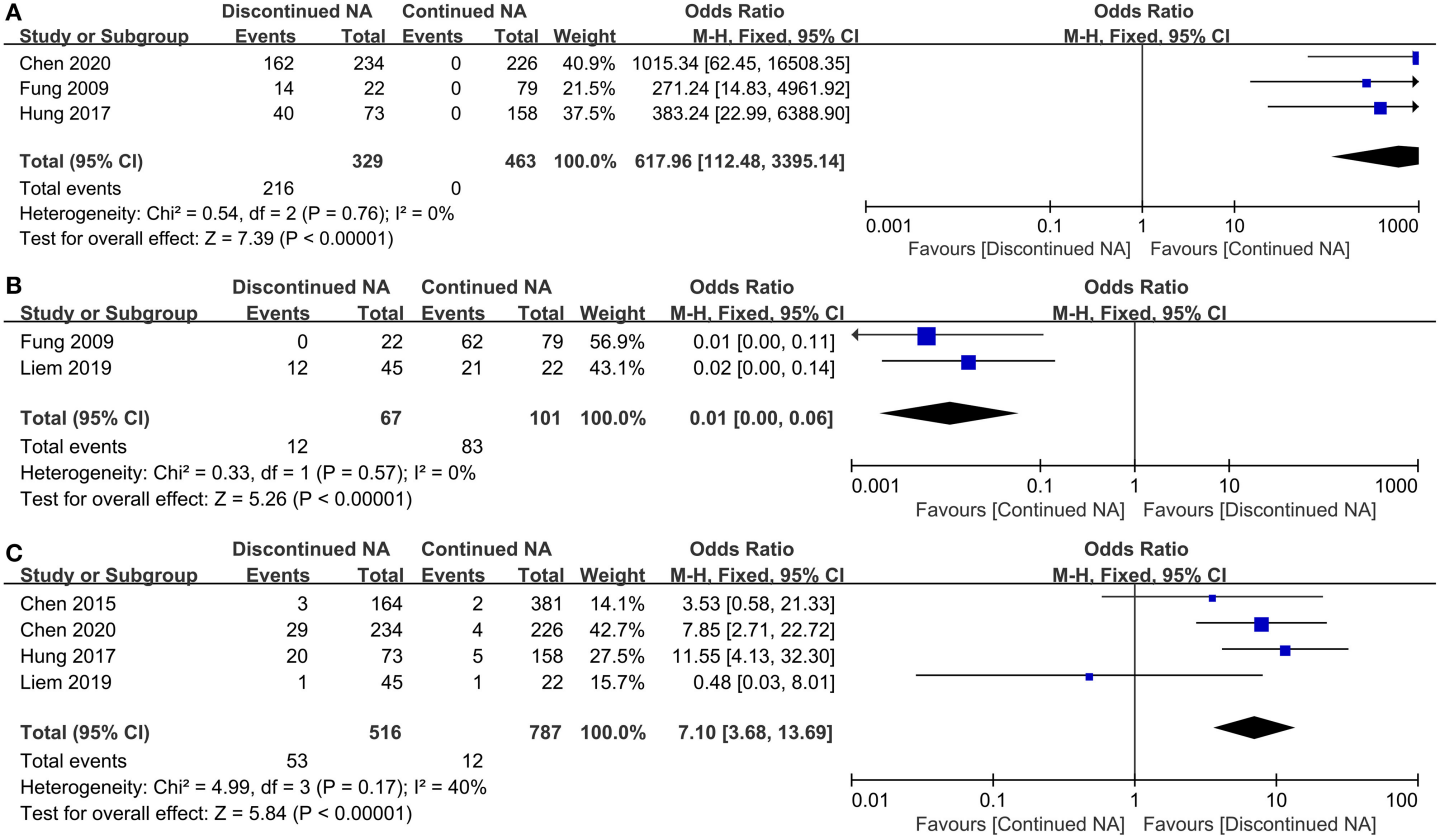
Forest plot on serological outcomes and virological outcomes comparing between continued Nucs therapy and discontinued Nucs therapy. **(A)** virological bound; **(B)** undetectable HBV DNA level; **(C)** HBsAg loss.

Virological and serological outcomes are depicted in Figure 3. Virtually three studies compared virological bound rate of CHB patients between Nucs off-therapy and continued groups. There is no heterogeneity (*I*^2^ = 0). Our result reveals that patients with continued Nucs treatment could preferably reduce the incidence of virological bound than those who discontinued Nucs therapy (OR = 617.96, 95% CI = 112.48–3,395.14, *p* < 0.001) (Figure 3A). In total, 12 CHB patients achieved undetectable HBV DNA levels in the off-therapy group, while 83 CHB patients accomplished undetectable HBV DNA levels in the continued group. The meta-analysis concluded that those who continued with Nucs treatment had a greater reduction in HBV DNA than those who stopped treatment (*p* < 0.001) (Figure 3B). In total, 53 CHB patients (among 516 CHB patients) experienced HBsAg loss in the Nucs off-therapy group, whereas 12 CHB patients (among 787 patients) experienced HBsAg loss in the continued group. According to estimated pooled OR, patients in Nucs off-therapy group exhibited a greater post-treatment HBsAg loss than those in the continued group (OR = 7.10, 95% CI = 6.68–13.69, *p* < 0.001) (Figure 3C).

### HCC Development

**Figure 4:**
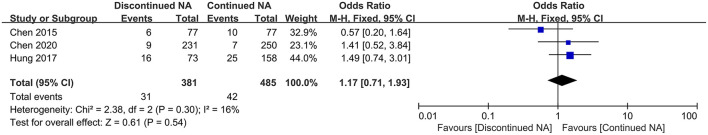
Forest plots on incidence of hepatocellular carcinoma comparing between continued Nucs therapy and discontinued Nucs therapy.

Three studies reported the long-term rate of HCC development in the Nucs off-therapy group compared to the continued Nucs group among CHB patients. In general, 8.67% of patients developed HCC in the off-therapy group during follow-up time, and 8.66% of patients developed HCC in the continued group. No statistically significant difference existed in HCC development between the two groups in all patients (OR = 1.17, 95% CI = 0.71–1.93, *p* = 0.54) (Figure 4).

### Publication Bias and Sensitivity Analysis

Considering that these eligible papers were <10, Begg's and Egger's tests for publication bias and sensitivity analysis were not used for the meta-analysis due to the low efficiency of qualitative and quantitative tests.

## Discussion

In contrast to “lifelong therapy” strategy, the field explores whether some patients with long-term Nucs therapy may be able to successfully withdraw Nucs therapy. Studies of stopping Nucs therapy remain limited, owing to concerns about virological rebound, ALT flare-up, risk of hyperbilirubinaemia, and liver failure after cessation of Nucs off-therapy. Our findings indicate that patients who discontinue Nucs therapy while being monitored have a greater risk of ALT flares-up than those who continue Nucs therapy. However, in three included studies, the definitions of ALT flare-up are different. In a study by Fung et al. (22), the ALT flare-up is defined as >2 ULN; in a study by Liem et al. (15), the ALT flare-up is defined as >5 times ULN; in a study by Chen et al. (14), patients experienced severe ALT flare-up that is more than 20 times ULN. Further research is needed to unify the ALT flare-up threshold. Simultaneously, Nucs off-treatment under the monitor increases the incidence of virological bound and results in a higher HBV DNA level than CHB patients who continued Nucs therapy because Nucs are inhibitors of hepatitis B virus (HBV) DNA polymerase and directly block the viral replication (23). However, it is difficult to completely eliminate viruses since Nucs are thought to have almost no influence on HBV cccDNA level in hepatocytes, representing the key HBV replicative intermediate (24). Intrahepatic cccDNA serves primarily as a template for viral RNA transcription, which results in the development of offspring virion DNA (24). In this long-term state, it is unlikely that patients would benefit from continuing to receive Nucs therapy; therefore, some researchers proposed discontinuing Nucs therapy for CHB patients who were closely monitored (14). The prospective studies have indicated no increased risks of hyperbilirubinaemia (14, 15) or hepatic decompensation (13, 14) in CHB patients undergoing Nucs off-treatment than those in the Nucs continued group under the monitor. Moreover, our meta-analysis results are consistent with them. There is also the possibilty of type 2 statistical errors. Therefore, it is vital for future research to increase the number of the included subjects. Some evidence suggests that virological relapse after Nucs off-therapy may induce immune activation with chemokines/cytokines as well as T cell responses (25), although additional research is required to support this.

Moreover, a previous study (14) showed that 12 and 2 patients experienced severe ALT flares-up and hepatic decompensation after Nucs off-therapy, respectively. No patient died after timely re-treatment. Therefore, it is critical to emphasize the importance of meticulous patient follow-up to diagnose as early as possible any ALT flares associated with an increased risk of liver failure, particularly in liver cirrhosis patients, to rapidly restart antiviral therapy. As a minimum, the study recommends monitoring liver function tests at week 6, week 12, week 18, and week 24, and then every 3 months for the first 2 years. Notably, potential safety concerns when ALT concentrations increase mean that restarting therapy should be considered (26).

HBsAg seroclearance is rare during Nucs treatment (27). Nucs have potent antiviral activity but do not have a direct immunomodulatory effect. One key finding in our study was that CHB patients who discontinued the Nucs therapy were more likely to develop HBsAg loss than those who continued Nucs therapy (28, 29), similar to findings observed in previous prospective studies (13, 15). Although host HBV-specific T-cell immunity may be modulated and recovered during long periods of HBV suppression via Nucs treatment (30), HBV relapse after Nucs off-therapy may trigger efficient immunological response that enhances the responsiveness of HBV-specific T cells and NK cells function. However, a recent study was inconsistent with it. The study indicated no statistically significant difference in HBsAg loss after discontinuing Nucs treatment compared to continued Nucs therapy. This may be because ALT flares can also precede HBsAg loss; therefore, restarting treatment could potentially prevent patients from achieving this positive outcome (HBsAg loss)(26).

Regarding the association between HBV genotypes and HBsAg seroclearance, a previous study (31, 32) found no statistically significant differences in HBsAg seroclearance rates among the different genotypes. Numerically, genotype A appeared to have the highest HBsAg seroclearance rate, and genotype F had the lowest, but the number of studies and patients with genotypes A, D, and F were small. Further analysis comparing genotype B and C patients also found no significant difference in HBsAg seroclearance rate (32, 33). However, a study (34) suggests that patients with genotype B had a higher rate of HBsAg seroclearance than those with genotype C, and yet a previous study (35) suggesting that genotype C was associated with a higher lifetime chance of HBsAg loss than genotype B. Due to lack of sufficient data, we cannot investigate the relationship between HBsAg clearance and HBV genotype in our meta-analysis. Consequently, there is a need to further compare the incidence of HBsAg loss among CHB patients between discontinued and continued Nucs therapy groups in future well-designed studies.

HCC is a major cause of liver-related mortality in CHB patients; however, one of the primary functions of antivirus is to prevent HCC (36). Previous studies have demonstrated that long-term Nucs therapy exhibited a reduced incidence of HCC (37, 38). It is unknown whether Nucs therapy cessation would have a detrimental impact on reducing HCC incidence. In our meta-analysis, Nucs off-therapy did not exhibit an increased risk of HCC than the Nucs-continued group among CHB patients under the monitor, consistent with a study from Taiwan (14). Simultaneously, the result should be likewise interpreted with caution due to limited sample size of the study. In addition, there may be insufficient follow-up time because HCC development is a long-term outcome (39). Therefore, our consequence still requires further validation. Despite this, Hall et al. reported a study that recommends restarting Nucs therapy in patients with persistent HBV DNA levels >2,000 IU/ml after stopping Nucs therapy, regardless of ALT level, to minimize the risk of HCC over time (36).

In addition, the 2017 EASL recommendations also suggested that Nucs may be discontinued in selected non-cirrhotic HBeAg-negative CHB patients with long-term (≥3 years) on-therapy virological suppression who will remain under close post-Nucs therapy follow-up (40). AASLD guidelines still do not recommend Nucs discontinuation in HBeAg-negative CHB patients with long-term virological and biochemical remission (41). As a result, more comprehensive studies are required to evaluate the effect of Nucs off-therapy in CHB patients.

A significant strength of this research is that it is the first meta-analysis to compare long-term outcomes, including biochemical, serological, and virological outcomes, as well as HCC development rate between CHB patients who discontinued and maintained Nucs therapy. However, it still requires evaluation for Nucs off-therapy safety among CHB patients under the monitor further. The meta-analysis should be interpreted because of certain limitations. Firstly, due to the difference of definitions of ALT flare-up in three included studies, further research is needed to unify the ALT flare-up threshold. Secondly, the sample size included was insufficient due to a limited number of related studies; therefore, publication bias and sensitivity analysis were not allowed to conduct further, affecting the last conclusions. Thirdly, we were unable to acquire unpublished articles, and the language was confined to English merely. Then, off-Nuc studies which only with discontinued group (without contrast) do not conform to our included criteria, but it may underestimate the off-Nuc clinical events. Finally, most of the included literature was from Chinese studies, which lacked certain representativity and could not be widely popularized.

In conclusion, Nucs off-therapy can cause virological relapse and liver enzyme damage without increasing liver failure risk under the monitor compared with those who continued therapy among CHB patients. In the long-term, Nucs off-therapy did not show an increased risk of developing HCC compared to the Nucs therapy group. Importantly, patients who discontinue therapy may exhibit a higher chance of achieving HBsAg loss than those who continue Nucs therapy. Although our study suggests that Nucs off-therapy may have potential benefits for CHB patients under the monitor, we must emphasize that this might cause severe clinical consequences. Hence, it requires close monitoring after Nucs off-therapy and timely restarting of Nucs therapy when ALT concentrations increase. Further, larger and well-designed studies are required to evaluate Nucs off-therapy safety.

## Data Availability Statement

The original contributions presented in the study are included in the article/Supplementary Material, further inquiries can be directed to the corresponding author/s.

## Author Contributions

LY designed the research process. MW and DY searched the database for corresponding articles. RF and NW extracted useful information from the articles above. XS used statistical software for analysis. MW and MQ drafted the meta-analysis. YZ and DY polished this article. All authors had read and approved the manuscript and ensured that this was the case.

## Conflict of Interest

The authors declare that the research was conducted in the absence of any commercial or financial relationships that could be construed as a potential conflict of interest.

## Publisher's Note

All claims expressed in this article are solely those of the authors and do not necessarily represent those of their affiliated organizations, or those of the publisher, the editors and the reviewers. Any product that may be evaluated in this article, or claim that may be made by its manufacturer, is not guaranteed or endorsed by the publisher.

## Abbreviations

CHB: chronic hepatitis B
IFN: interferon
Nucs: nucleos(t)ide analogs
HCC: hepatocellular carcinoma
HBeAg: hepatitis B “e” antigen
HBsAg: hepatitis B surface antigen
HBV: hepatitis B virus
ALT: alanine aminotransferase
OR: odds ratio
95%CI: 95% confidence interval
RCTs: randomized controlled trials
PRISMA-P: preferred reporting items for systematic review and meta-analysis protocols
NOS: newcastle-ottawa quality assessment scale
ULN: upper limit of normal.
