# Supplementary material for: The Impact of Nucleos(t)ide Analogs Off-Therapy Among Chronic Hepatitis B Patients: A Systematic Review and Meta-Analysis
Source: Front Public Health. 2021 Sep 10;9:709220. doi: 10.3389/fpubh.2021.709220 (PMC8460900; doi:10.3389/fpubh.2021.709220)
Supplement: Supplementary file 1 [file Table_1.DOCX]

**Supplementary Table 1.** Quality assessment of studies included.

| Author, year,  Study (RCT) | Sequence  Generation | | Allocation  Concealment | | Blinding | Incomplete  outcome data | | Selective  outcome reporting | | Free of  other bias |  |  |  |  |  |
| --- | --- | --- | --- | --- | --- | --- | --- | --- | --- | --- | --- | --- | --- | --- | --- |
| Liem, 2019 | low risk | | low risk | | low risk | low risk | | low risk | | unclear risk |  |  |  |  |  |
|  | | | | | | | | | | |  |  |  |  |  |
| Author, year,  Study (Observational)POBS | | **Selection (Out of 4)** | | | | | | | | | **Comparability**  **(Out of 2)** | **Outcomes (Out of 3)** | | | **Total**  **(Out of 9)** |
|  |  | Representativeness of exposed cohort | | Selection of nonexposed cohort | | | Ascertainment  of exposure | | Outcome not present at the start of the study | |  | Assessment of outcomes | Length of follow-up | Adequacy of follow-up |  |
| Fung, 2009 | | 1 | | 1 | | | 1 | | 1 | | 1 | 1 | 1 | 1 | 8 |
| Chen, 2015 | | 1 | | 1 | | | 1 | | 1 | | 2 | 1 | 1 | 1 | 9 |
| Hung, 2017 | | 1 | | 1 | | | 1 | | 1 | | 1 | 1 | 1 | 1 | 8 |
| Chen, 2020 | | 1 | | 1 | | | 1 | | 1 | | 2 | 1 | 1 | 1 | 9 |

The RCT and observational studies were assessed by the Cochrane Collaboration’s tool and Newcastle-Ottawa Quality Assessment Scale, respectively.

Risk of bias was assessed as “low risk”, “high risk” or “unclear risk”.
